# Alternative Plant Vitrification Solution A3-80% and Initial Ammonium-Free Regrowth Medium Enable Cryobanking of Chrysanthemum Germplasm

**DOI:** 10.3390/plants12051059

**Published:** 2023-02-27

**Authors:** Hyoeun Lee, Junsun Park, Sang-Un Park, Haenghoon Kim

**Affiliations:** 1 Department of Agricultural Life Science, Sunchon National University, Suncheon 57922, Republic of Korea; 2 Department of Crop Science, Chungnam National University, Daejeon 34134, Republic of Korea

**Keywords:** C4-35%, droplet-vitrification, liquid overlay, shoot tips, two-step preculture, three-step regrowth

## Abstract

Cryopreservation, storing biological material in liquid nitrogen (LN, −196 °C), offers a valuable option for the long-term conservation of non-orthodox seeds and vegetatively propagated species in the sector of agrobiodiversity and wild flora. Although large-scale cryobanking of germplasm collections has been increasing worldwide, the wide application of cryopreservation protocol is hampered by a lack of universal cryopreservation protocols, among others. This study established a systematic approach to developing a droplet-vitrification cryopreservation procedure for chrysanthemum shoot tips. The standard procedure includes two-step preculture with 10% sucrose for 31 h and with 17.5% sucrose for 16 h, osmoprotection with loading solution C4-35% (17.5% glycerol + 17.5% sucrose, *w/v*) for 40 min, cryoprotection with alternative plant vitrification solution A3-80% (33.3% glycerol + 13.3% dimethyl sulfoxide + 13.3% ethylene glycol + 20.1% sucrose, *w/v*) at 0 °C for 60 min, and cooling and rewarming using aluminum foil strips. After unloading, a three-step regrowth procedure starting with an ammonium-free medium with 1 mg L^−1^ gibberellic acid (GA_3_) and 1 mg L^−1^ benzyl adenine (BA) followed by an ammonium-containing medium with and without growth regulators was essential for the development of normal plantlets from cryopreserved shoot tips. A pilot cryobanking of 154 accessions of chrysanthemum germplasm initiated with post-cryopreservation regeneration of 74.8%. This approach will facilitate the cryobanking of the largest Asteraceae family germplasm as a complementary long-term conservation method.

## 1. Introduction

Chrysanthemum (*Dendrathema grandiflourum*) is one of the major ornamental crops worldwide. It belongs to the Asteraceae (Compositae) family, which is the most prominent, consisting of over 32,900 species of horticultural and medicinal crops and wild species [1]. Cryopreservation, storing biological material in liquid nitrogen (LN, −196 °C), provides a valuable option for the long-term preservation of plant genetic resources [2]. As a complementary approach to seed genebank and field genebank, in vitro culture and cryopreservation are potent tools for safe backup germplasm collections of vegetatively propagated crops [2,3]. Cryopreservation of shoot tips, as a somatic tissue, is the preferred option for the seed-producing species of heterozygotes, such as most fruits, flowers, and some vegetables, for the interest of genetic stability. Cryopreservation of short-lived seed species and vegetatively propagated species is of importance since it is considered the only option for long-term conservation strategy [2,3].

The triangle of cryopreservation would be plant material, protocol, and manipulation skills [3]. Numerous studies indicated genotype-dependent responses [4] and the importance of healthy donor plants for cryopreservation [2,5,6]. The protocol is the central part of the studies, and the droplet-vitrification (DV) is a multiple-stage procedure: pre-LN (preculture, osmorotection with loading solution, cryoprotection with vitrification solution), LN (cooling in LN, warming, unloading), and post-LN (regrowth) [3]. However, most investigations have focused on optimizing the pre-LN stages, such as preculture (sucrose concentration, duration) and cryoprotection (PVS2 duration) [2,7]. The most common treatment conditions are preculture with 10% sucrose and cryoprotection with Plant Vitrification Solution 2 (PVS2) [2,7]. In addition to determining each stage, tunning and balancing the whole process is essential. Moreover, standard underoptimized conditions may lead to erroneous results, such as our previous study using *P. yatabeanus* [8], since we may obtain lower regrowth regardless of the treatment conditions.

Most of the literature using the solution-based vitrification methods investigated singular-step sucrose preculture [2,7]. The most frequent option is 10% sucrose (0.3 M) for 1–3 days [2,7]. Optimizing step-wise sucrose preculture is a prerequisite for adapting to the osmotic stress induced by highly concentrated vitrification solutions [9,10]. The selection of a proper Plant Vitrification solution (PVS) is crucial since it protects the explants from freezing injury while injuring them by osmotic stress and chemical cytotoxicity [11]. Hence, the balancing of cryoprotection and cytotoxicity is essential when we select the appropriate PVSs. Kim et al. [12] designed alternative PVSs, and A3-90% produced 19.7~39.0% higher LN regrowth over PVS2 in diverse species of organized tissue (shoot tips), among others [12,13]. In contrast, diluents are the preferred option for tiny or undifferentiated tissue. In this case, the alternative PVS was slightly effective compared to PVS2: A3-80%, +4.7% (no significance) for embryogenic callus [14]; A3-70%, +10.5% for hairy roots [15]. It reflects that PVS2 was designed for callus [16] and is powerful for undifferentiated cells and meristem-size tiny tissues.

Manipulation of mother plantlets and preparation of explants also need comprehensive skills. Our recent study indicated vigorous growth of donor plants supported by liquid overlay on the gelled medium was critical for the normal regeneration of cryoprotected control (LNC) and cryopreserved (LN) shoot tips [17]. Moreover, the initial ammonium-free regrowth medium for five days was beneficial for coping with ammonium-induced oxidative stress [18]. Teixeira da Silva et al. [19] reviewed chrysanthemum cryopreservation and recommended using PVS3 instead of PVS2 and its alternatives. Some or most cryopreserved shoot tips were regenerated via a callus-like lag phase using a conventional regrowth with an ammonium-containing regrowth medium, regardless of transferring to a new medium, in chrysanthemum [10,19,20,21,22] and other Asteraceae species [23]. Even though explants survived freezing, diverse morphogenetic responses were reported in cryopreserved shoot tips, such as single shoots, multiple shoots, deformed hyperhydrated shoots, no or retarded shoot elongation, and reduced chlorophyll content in leaves [21,22,24,25].

This study investigated preculture, alternative PVSs, cooling/warming containers, and the effect of ammonium-ion and growth hormones in a regrowth medium. It highlights the usefulness of an alternative PVS A3-80% and the importance of an initial ammonium-free regrowth medium for normal regeneration of cryopreserved shoot tips. The pilot implementation of cryobanking for chrysanthemum collection was initiated using the optimized protocol.

## 2. Results

### 2.1. Optimization of Droplet-Vitrification Procedure for Chrysanthemum

#### 2.1.1. Effect of Pre-LN Conditions on Post-Cryopreservation Recovery

**Figure 1:**
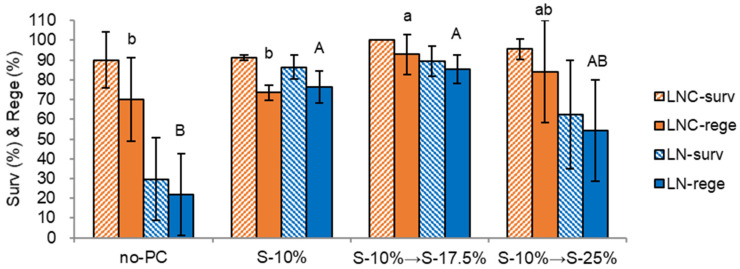
Effect of preculture conditions on survival (surv) and regeneration (rege) of cryoprotected control (LNC) and cryopreserved (LN) chrysanthemum var. ‘Little rock’ shoot tips. no-PC, non preculture; S-10%, preculture with 10% sucrose for 24 h; S-10% → S-17.5%, 10% sucrose 31 h → S-17.5% 16 h; S-10% → S-25%, 10% sucrose 16 h → S-25% 16 h. Standard condition (S-10% → S-17.5%): two-step preculture 10% sucrose (S-10%) for 31 h and S-17.5% for 16 h, osmoprotectant with C4-35% for 40 min, cryoprotected with A3-80% ice for 60 min, cooling, and warming using aluminum foil strips, thawing with S-35% solution for 30 s (40 °C) and unloading for 40 min, three-step regrowth with RM1 (ammonium-free MS)-RM2-MSF. Means with the same letters (a,b and A,B) in each graph are not significantly different by Least Significant Difference (LSD) Test (*p* < 0.05) using SAS.

The sucrose concentration in two-step preculture solutions significantly affected the post-LN survival and regeneration. A step-wise preculture with 10% sucrose (S-10%) for 31 h and 17.5% sucrose (S-17.5%) for 16 h produced the highest LN survival and regeneration (89.3% survival and 86.7% regeneration), which was higher than the singular S-10% for two days (S-10%, 86.5% survival, and 76.3% regeneration) (Figure 1). In contrast, non-preculture (no-PC) and higher sucrose concentrations of S-10% → S-25% resulted in the lowest regeneration of 22.0% for no-PC and 43.4% for S-10% → S-25%. Although chrysanthemum shoot tips are sensitive to osmotic stress, they can adapt to dehydration tolerance by proper step-wise preculture.

**Figure 2:**
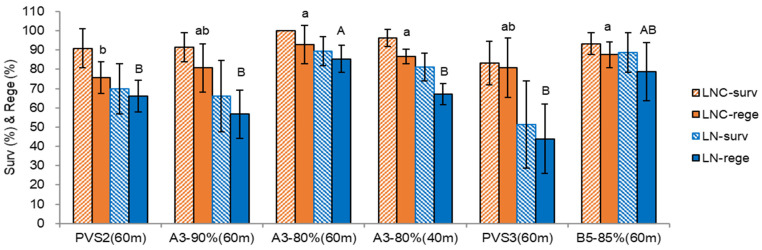
Effect of plant vitrification solutions on survival (surv) and regeneration (rege) of cryoprotected control (LNC) and cryopreserved (LN) chrysanthemum var. ‘Little rock’ shoot tips. PVS2 (60 m), cryoprotection with PVS2 for 60 min at 0 °C; A3-90% (60 m), A3-90% for 60 min at 0 °C; A3-80% (60 m), A3-80% for 60 min at 0 °C; A3-80% (40 m), A3-80% for 40 min at 0 °C; PVS3 (60 m), PVS3 for 60 min at 25 °C; B5-85% (60 m), B5-85% for 60 min at 25 °C. Means with the same letters (a,b and A,B) in each graph are not significantly different by LSD Test (*p* < 0.05) using SAS.

The plant vitrification solutions (PVSs) significantly affected the post-cryopreservation recovery of chrysanthemum shoot tips (Figure 2). Among the PVSs tested, A3-80% for 60 min produced the highest survival and regeneration of both LNC and LN shoot tips. The chrysanthemum shoot tips were sensitive to the cytotoxicity of PVS2 (A1-73.7%), A3-90% (alternative PVS), and PVS3 (B1-100%). A diluent of A3-90%, A3-80%, and a diluent of PVS3, B5-85% produced higher survival and regeneration of both LNC and LN shoot tips than the original PVSs. A shorter duration with A3-80% for 40 min (A3-80% (40 m)) produced lower LN survival and regeneration (LN survival 79.2%, regeneration 64.4%) compared to the standard condition of 60 min (A3-80% (60 m), LN survival 89.3%, regeneration 86.7%). This study indicates that the chrysanthemum shoot tips are sensitive to chemical toxicity and osmotic stress of the highly concentrated PVSs. Therefore, their diluents can be an alternative option in such a case.

**Figure 3:**
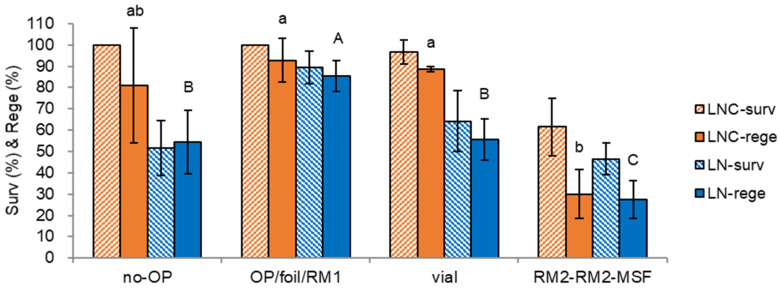
Effect of osmoprotection (OP), cooling/warming container, and regrowth medium on survival (surv) and regeneration (rege) of cryoprotected control (LNC) and cryopreserved (LN) chrysanthemum var. ‘Little rock’ shoot tips. no-OP, non-osmoprotection; OP/foil/RM1, as a standard condition, osmoprotection with C4-35% for 40 min, cooling/warming using aluminum foil strips, regrowth in RM1 (ammonium-free MS)-RM2-MSF medium; vial, cooling/warming using 2 mL cryovial; RM2-RM2-MSF, three-step regrowth with RM2 (ammonium-containing standard MS)-RM2-MSF. Other conditions of each treatment were the same as the standard condition. Means with the same letters (a,b and A–C) in each graph are not significantly different by LSD Test (*p* < 0.05) using SAS.

We tested other options of no-osmoprotectant, cooling/rewarming containers, and regrowth medium. Similar to the no-PC in Figure 1, non-osmoprotection (no-OP in Figure 3) also was stressful to the shoot tips and eventually resulted in lower LN survival and regeneration (37.7% and 32.2% lower) compared to the standard condition of OP-treated shoot tips (OP/foil/RM1, 89.3% survival, 86.7% regeneration). Despite osmotic sensitivity, the shoot tips acquired the adaptation by the step-wise sucrose preculture. In addition to the osmotic stress, insufficient cryoprotection may cause lower LN regeneration during the cooling and rewarming stage when the cryovial (vial) was used as a cooling container. Regrowth with an initially ammonium-containing regrowth medium (RM2-RM2-MSF) produced the lowest survival and regeneration for LNC and LN shoot tips. This result implies cryoprotection with A3-80% was stressful to shoot tips, resulting in ammonium-induced oxidative stress and, finally, marginal survival and regeneration of LNC and LN shoot tips.

#### 2.1.2. Effect of Regrowth Media in Three-Step Regrowth Procedure

**Figure 4:**
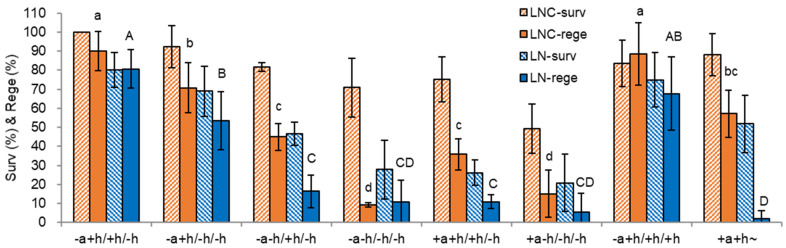
Effect of ammonium nitrate and growth regulators in three-step regrowth procedure on survival (surv) and regeneration (rege) of cryoprotected control (LNC) and cryopreserved (LN) chrysanthemum var. ‘Little rock’ shoot tips. step 1/step 2/step 3; Step 1 was performed on MS medium with (+) or without (−) ammonium nitrate and growth hormones (1 mg L^−1^ GA_3_ + 1 mg L^−1^ BA) in dark for five days. Steps 2 and 3 were performed on MS medium containing ammonium nitrate and with (+) or without (−) growth hormones (1 mg L^−1^ GA_3_ + 1 mg L^−1^ BA) under light, 40 µE m^−2^ s^−1^ for 16 and 14 days, respectively. standard condition (−a+h/+h/−h, treatment 1): two-step preculture 10% sucrose (S-10%) for 31 h and S-17.5% for 16 h, osmoprotection with C4-35% for 40 min, cryoprotected with S3-80% ice for 60 min, cooling and warming using aluminum foil strips, thawing with S-35% solution for 30 s (40 °C) and unloading for 40 min, three-step regrowth with RM1 (ammonium-free MS)-RM2-MSF. Means with the same letters (a–d and A–D) in each graph are not significantly different by Least Significant Difference Test (*p* < 0.05).

We further investigated the combinational effects of with or without (+ or −) ammonium (a) and plant growth hormones (h) in the regrowth medium at steps 1, 2, and 3 for 5, 16, and 14 days, respectively (step 1/step 2/step 3, Figure 4). We found the combination of an initial ammonium-free medium and growth hormones in three-step regrowth media is a critical factor no less than any pre-LN stages and conditions tested in Figure 1, Figure 2 and Figure 3. The highest survival and regeneration were observed when the cryoprotected control (LNC) and cryopreserved (LN) shoot tips were regrown with the standard condition (−a+h/+h/−h). It employs step 1 (−a+h) on an ammonium-free medium with growth hormones (1 mg L^−1^ gibberellic acid (GA_3_) and 1 mg L^−1^ benzyl adenine (BA)) followed by step 2 (+h) using an ammonium-containing medium with the same growth hormones and step 3 (−h) on an ammonium-containing medium without growth hormones.

The second-and third-best options (−a+h/+h/+h, −a+h/−h/−h) contained the same treatments of ammonium-free and with growth hormones (−a+h/) in step 1 with the only difference of including (/+h/+h) or excluding (/−h/−h) growth hormones at step 2 and 3. All other treatments, i.e., ammonium-containing (+a/) or ammonium-free with the absence of growth hormones (−a−h/) in step 1, resulted in marginal regeneration of LNC (9.2~35.8%) and LN (5.6~10.8%) shoot tips. A regrowth medium containing ammonium and growth regulators failed to regenerate without transferring to a new medium (+a+h~).

Overall, ammonium-free in step 1 and growth regulators in steps 1 (more important) and 2 are critical for normal regeneration of LNC and LN shoot tips. It reflects the nature of the toxic effect of ammonium-ion in step 1 and the beneficial effect of growth hormones in steps 1 and 2. The absence of growth hormones in step 3 is helpful for the normal regeneration of shoot tips.

### 2.2. Cryobanking of Chrysanthemum Germplasm

After developing the droplet-vitrification protocol, a pilot cryobanking started in collaboration with the Flowers Breeding Research Institute (FBRI) of Gyeongsangnam-do Agricultural Research & Extension Services (GARES), one of the host institutes of chrysanthemum germplasm under the national germplasm management program.

In vitro plantlets were multiplicated via nodal sections through the sequential subcultures. Node-cutting-derived shoot tips were subjected to standard droplet-vitrification. A total of 154 accessions of three types of standard, spray, and pot varieties were stored in liquid nitrogen (LN); average LN survival was 84.9%, and LN regeneration was 74.8% (Table 1). The range of LN regeneration for each type was from 44.4~50.0% to 94.4~100%, whereas no significant differences were noticed among types of standard, spray, and pot varieties.

**Figure 5:**
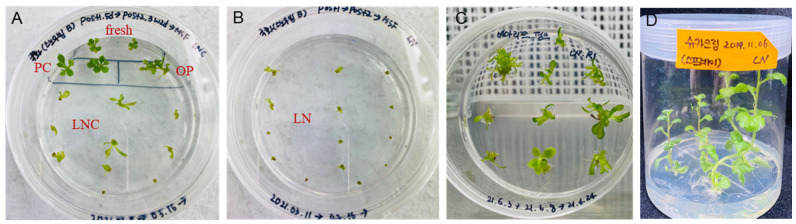
Regrowth of cryopreserved chrysanthemum three varieties in three-step regrowth of RM1 (ammonium-free MS)-RM2-MSF. Regrowth of var. ‘Snow beam’ shoot tips of fresh, PC, OP, and LNC (**A**) and LN (**B**) after one week. Regrowth of var. ‘Bializz pink’ LN shoot tips after four weeks (**C**). Regeneration of var. ‘Sugar cream‘ shoot tips after eight weeks (**D**). Fresh, fresh-control shoot tips; PC, preculture with 10% sucrose for 31 h and 17.5% sucrose 16 h; OP, PC and osmoprotected with C4–35% for 40 min; LNC, PC-OP and cryoprotected (CP) with A3–80% ice for 60 min but not cryopreserved; LN, PC-OP-CP, and cryopreserved (LN). LNC and LN shoot tips were unloaded with 30% sucrose solution for 40 min before regrowth.

Figure 5 shows a random sample photograph of three cryopreserved chrysanthemum varieties. Shoot tips of one of the low-LN-regeneration var. ‘Snow beam’ regrow slowly compared to the high-LN regeneration varieties (Figure 5A,B). Another median-LN-regeneration var. ‘Bializz pink’ showed mild hyperhydration after four weeks of regrowth (-C). While complete regeneration was observed in var. ‘Sugar cream’ (-D).

Among the cryopreserved accessions, some varieties, such as var. ‘Purple pearl’ (44.4%) and var. ‘Orange cap’ (50.0%) showed relatively lower LN regeneration. We identified that these accessions were slowly grown during subcultures, owing to the delayed subculture inoculation and other unidentified reasons. For revitalizing the donor plantlets, we inoculated the apical sections, instead of nodal sections, as planting material and applied a liquid overlay on the gellan gum-gelled medium 10–14 days after inoculation.

**Figure 6:**
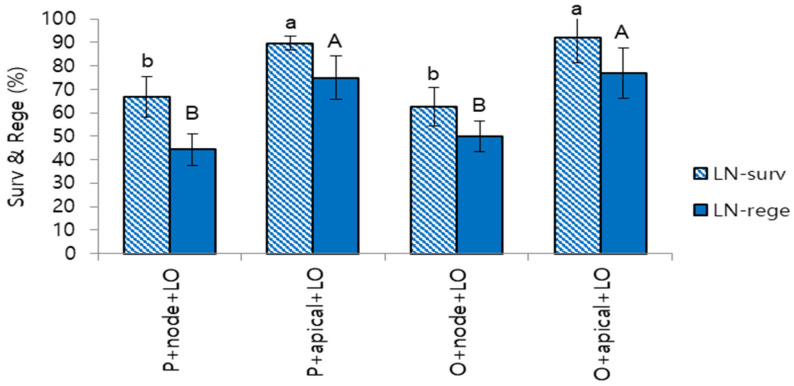
Effect of the revitalization of donor plantlets during subcultures on survival (surv) and regeneration (rege) of cryopreserved (LN) chrysanthemum shoot tips. P+node+LO, var. ‘Purple pearl’, inoculating nodal sections + liquid overlay on the gelled medium. P+apical+LO, var. ‘Purple pearl’, inoculating apical sections + liquid overlay on the gelled medium. O+node+LO, var. ‘Orange cap’, inoculating nodal sections + liquid overlay on the gelled medium. O+apical+LO, var. ‘Orange cap’, inoculating apical sections + liquid overlay on the gelled medium. Means with the same letters (a,b and A,B) in each graph are not significantly different by Least Significant Difference Test (*p* < 0.05).

LN survival and regeneration of these varieties increased after 1–2 cycles of subculture using apical section + liquid overlay. LN regeneration was increased from 44.4% to 75.0% for var. ‘Purple pearl’ (30.6% increase), from 50.0% to 76.9% for var. ‘Orange cap’ (26.9% increase) (Figure 6). This result implies careful manipulation by maintaining plantlets’ vigor during subcultures is crucial in cryobanking stages.

## 3. Discussion

### 3.1. Development of Droplet-Vitrification Protocol

A singular-step preculture with 17.5% sucrose (0.5 M) was detrimental in the preliminary investigation of this study (data not shown). This study indicates the two-step preculture with 10% sucrose and 17.5% sucrose is superior to other options. The osmolarity of meshed nodal sections of in vitro-grown plantlets was similar to that of 10% sucrose (Osm 0.42). Hence, 1–2 day preculture with 10% sucrose may induce mild adaptation by several physiological changes [26,27]. A 10% sucrose is suitable for susceptible wild species [8,28]. A higher sucrose concentration is preferred if explants endure higher concentration: 17.5% sucrose for most species [29,30,31,32] and 25% for some tolerant materials, such as potato [5]. A three-step preculture with 10% sucrose 31 h → 17.5% 17 h → 25% 7 h was suitable for inducing osmotolerance and thus produced the highest LN regeneration in chrysanthemum [10]. This appropriated sucrose preculture substituted the effect of cold acclimation [19].

Chrysanthemum shoot tips were sensitive to the chemical toxicity of PVS2 (73.7% *w/v*) and its alternative PVS A3-90% [12]. Hence, A3-80%, a dilution of A3-90%, produced the highest LN regeneration (86.7%) among the PVSs tested. A3-80% contains a 3.4% lower concentration of permeating cryoprotectants ((dimethyl sulfoxide (DMSO) and ethylene glycol (EG)). A 9.7% higher semi-permeating and non-permeating cryoprotectants (glycerol and sucrose) than PVS2. Hence, it can be less toxic chemically and more stressful osmotically compared to PVS2. Therefore, with proper step-wise preculture, A3-80% can dehydrate the samples with less chemical cytotoxicity. Both shorter 40 min with A3-80% (A3-80% (40 m)) or lower concentrations (A3-70%) for 60 min (data not presented) resulted in lower LN survival and regeneration, indicating shoot tips were insufficiently cryoprotected. Chrysanthemum is also sensitive to the osmotic stress of PVS3 (100% *w/v*). Thus, B5-85%, dilution of PVS3 to 85% *w/v*, obtained significantly higher LN survival and regeneration over PVS3. This study highlights the usefulness of alternative PVSs for sensitive materials. With elevated glycerol and sucrose concentration from the PVS2, A3-90% (37% glycerol + 15% DMSO + 15% EG + 22.5% sucrose) tends to facilitate efflux of water and influx of cryoprotectants compared to PVS2 and thus produced higher LN regeneration in organized shoot tips [12,13].

In osmotic stress conditions, chrysanthemum shoot tips showed lower LN regeneration; no-PC 22.0%, PVS3 43.9%, S-10% → S-25% 54.3%, no-OP 54.4%, A3-90% 56.7%. Regrowth with an initially ammonium-containing regrowth medium (RM2-RM2-MSF) also resulted in 27.6% LN regeneration. These stress conditions have a more considerable standard deviation than the optimum standard condition (preculture S-10% → S-17.5%, osmoprotection C4-35%, cryoprotection A3-80% 60 min, regrowth RM1-RM2-MSF), as seen in Figure 1, Figure 2, Figure 3 and Figure 4. The survival and regeneration of cryoprotected control (LNC) and cryopreserved (LN) shoot tips were drastically decreased when they were regrown with an ammonium-containing MS medium. This ammonium-induced oxidative stress happened with cryoprotection with PVS AS-80% on ice for 60 min rather than cooling and rewarming stages, as in the case of *P. yatabeanus* [18]. Using the aluminum foil strips (so-called ‘droplet-vitrification’ method) was superior to using cryovials (so-called ‘vitrification’ method) as cooling and rewarming containers when the cryoprotection was insufficient due to the cytotoxicity of PVSs, and hence faster cooling and rewarming rates in foil strips protect the shoot tips from crystallization and recrystallization, as in many species of structured shoot tips [2].

This study indicates that combining an ammonium-free medium and growth regulators in three-step regrowth is essential for the normal regeneration of LNC and LN shoot tips. More specifically, step 1 on an ammonium-free medium with 1 mg L^−1^ GA_3_ and 1 mg L^−1^ BA followed by step 2 using an ammonium-containing medium with the same growth regulators and step 3 on an ammonium-containing medium without growth regulators (−a+h/+h/−h in Figure 4, LNC 90.0%, LN 80.7%) was the best option. The ammonium-containing medium in step 1 or without growth regulators in steps 1 and 2 resulted in nill LNC and LN regeneration (LNC 9.2~35.8%, LN 5.6~10.8%). Conventional regrowth with an ammonium-containing medium with growth regulators without transfer to a new medium (+a+h~ in Figure 4) failed to direct regeneration. In this condition, the original LNC and LN shoot tips stopped regrowth and regenerated shoots after intermediate callus-like structures [10,19,20,21,22]. They sometimes showed diverse morphogenetic responses [21,22,24,25].

After optimizing the vitrification protocol, we can evaluate each factor. This study reveals that the regrowth medium (initial ammonium-containing medium and with growth hormones vs. initial ammonium-containing and without hormones) is the most critical factor. The second to fourth factors were preculture (10% sucrose → 17.5% sucrose vs. non-preculture), vitrification solutions (A3-80% vs. PVS3), and cooling and rewarming containers (foil strips vs. cryovial). It means that post-LN (regrowth medium) is even more crucial than the pre-LN stages (preculture, osmoprotection, cryoprotection) and LN (cooling and rewarming).

The intracellular ice crystallization and extensive cellular dehydration harm the samples in cryopreservation [33]. Osmotic stress and chemical toxicity [11] and reactive oxygen species (ROS)-induced oxidative stress [34,35] also decrease the recovery of LNC and LN shoot tips. The presence of ammonium may cause oxidative stress for plant materials experiencing severe stress during the pre-LN stages, especially cryoprotection with PVSs [36]. Hence, the initial ammonium-free regrowth medium increased LN regeneration by 17~61% over a traditional ammonium-containing medium [30,37]. Therefore, the primary strategy to cope with the hurdle of cryo-injury is to prepare the compatible material and to induce tolerance to the cytotoxicity induced by cryoprotection with highly concentrated PVSs in pre-LN stages. Following the LN, step-wise regrowth conditions need to be designed appropriately. It is assumed that ammonium-ion in the regrowth medium triggered oxidative stress which was induced during the cryopreservation procedures. ROS-induced oxidative stress was maximized during PVS treatment (LNC), LN exposure, and unloading [38]. Cryopreservation stress may reduce the metabolic activity of the explants, and the key enzymes of ammonia nitrogen metabolism could be inactivated or retarded after rewarming and unloading, leading to ammonium accumulation of toxic levels [39]. Hence, omitting ammonium in the regrowth medium for five days could support normal plant regeneration. Plant growth regulators (hormones) play a vital role in the organ development of LN plant materials [40,41]. Similar to our study, GA_3,_ in combination with cytokinins usually promotes direct plant formation from cryopreserved shoot tips [42].

### 3.2. Cryobanking of Germplasm

Implementation of cryobanking for germplasm conservation is a multidisciplinary work, including developing the standard protocol and applying it to a diverse range of plant materials [43]. Despite increasing cases of large-scale implementation of cryobanking [44,45], some critical challenges include protocol development, genotype-specific issues in adapting the protocols to multiple accessions, and a lack of skilled personnel [44]. The reason for genotype-specificity may be the sub-optimized protocol and insufficient protocol flexibility depending on the variation in the plant materials. With the same conditions, staff skills are a significant factor in the efficiency and uniformity of the results. It reflects the nature of cryopreservation not only as science and technology but also as art and technology. Reed et al. [46] considered skills transfer, training, and procedure interpretation as operational points in the international technology transfer of cryopreservation protocols.

The average LN regeneration of 154 accessions cryobanked as a pilot project was high (74.8%) regardless of the type of chrysanthemum cultivars tested. Two varieties (var. ‘Purple Pearl’ and var. ‘Orange cap’) showed lower LN regeneration (44.4% and 50.0%, respectively) in 2019 (the first year). We identified these accessions grew slowly, possibly due to the delayed inoculation of the subculture. As confirmed by Lee and Kim [17], inoculation of apical sections + liquid overlay for 1–2 subculture cycles improved LN regeneration by 30.6% and 26.9%. Reed et al. [46] also argued that plant health status and pre- and post-storage culture as non-cryogenic critical points. This study highlights the importance of maintaining the vigor of donor plantlets during the subcultures to prepare shoot tips. Inoculating apical sections and applying liquid overlay during the subcultures are helpful approaches to preparing healthy and vigorous donor plantlets [17]. In conclusion, cryobanking of chrysanthemum germplasm is a multidisciplinary work from designing suitable and vital plant material and developing optimized and flexible protocols to the manipulation skills of staff.

## 4. Materials and Methods

### 4.1. Plant Material, In Vitro Establish and Preparation of Mother Plants

For the development of the droplet-vitrification procedure, in vitro-grown chrysanthemum var. ‘Little rock’ was propagated using nodal segments (1.0~1.5 cm with one axillary bud) via repeated subcultures with hormone-free Murashige and Skoog media (MSF, [47]) with 30 g L^−1^ sucrose, 3.0 g L^−1^ gellan gum (KisanBio, Seoul, Republic of Korea), pH 5.8, in 300 mL SPL culture vessels (seven segments/vessel, SPL Life Sciences, Pocheon-si, Republic of Korea) at 25 °C under a 16/8 h light/dark photoperiod, 60 µE m^−2^ s^−1^, for six weeks (hereafter standard subculture). Liquid MSF medium (15 mL/vessel) was added to the gellan gum-gelled medium at day 10–14 (liquid overlay) to facilitate vigorous growth.

### 4.2. Optimization of Droplet-Vitrification Procedure

After repeated standard subcultures, shoot tips (1.5 mm) of var. ‘Little rock’ were excised from 6 to 7-day-old single nodal sections cultured on standard subculture media. Cryopreservation procedure was adopted by Lee et al. [10,18]. As a standard droplet-vitrification procedure, shoot tips were precultured in 10% sucrose (S-10%) for 31 h and 17.5% sucrose for 16 h at room temperature (RT), osmoprotected with C4-35% for 40 min at RT, and then cryoprotected with alternative PVS A3-80% on ice for 60 min. Then explants were placed in 5 µL of ice-cold A3-80% on aluminum foil strips (7 mm × 20 mm × 50 µm), plunged in LN for a minimum of 1 h. For rewarming, foil strips with shoot tips were transferred to 20 mL pre-heated (40 °C) unloading solution of 35% sucrose (S-35%) and kept for 40 min at RT, with the sucrose solution being replaced after the first 15 min. The preculture, osmoprotection, cryoprotection, and unloading solutions were made using MS basal media at pH 5.8. The solutions were filter-sterilized using a 0.45 µm membrane filter. The shoot tips retrieved from S-35% were blotted dry on sterilized filter paper and transferred to the regrowth medium 1 (RM1, NH_4_NO_3_–free MS medium + 1 mg L^−1^ GA_3_ + 1 mg L^−1^ BA, 30 g L^−1^ sucrose, 3.0 g L^−1^ gellan gum, pH 5.8) and cultured in the dark at 25 °C. After five days, explants were transferred to the RM2 medium (the same as RM1 except for NH_4_NO_3_) and cultured under 40 µE m^−2^ s^−1^ for 23 days. The developed shoots were transferred to the MSF medium for 14 days for normal regeneration.

In comparison with the standard condition of the droplet-vitrification procedure, diverse options at pre-LN (preculture, osmoprotection, vitrification solution; 10 conditions), LN (cooling/warming container; 1 condition), and post-LN (regrowth medium; 7 conditions) stages were investigated (Table 2). For instance, the treatment set of osmoprotection, cooling/warming container, and regrowth medium tested conditions of no-osmoprotection, osmoprotection, cryovial, and RM2 (ammonium-containing MS)-RM2-MSF. The treatment set of regrowth media investigated the combinational effect of ammonium (a) and plant growth hormones (h) in a regrowth medium using eight (standard + 7) options. During the experiment of each factor, other conditions remained the same as in the standard condition (indicated as “standard” in Table 2).

### 4.3. Cryobanking of Chrysanthemum Germplasm

For cryobanking of chrysanthemum germplasm, a bottle of in vitro plantlets or in vivo cuttings was introduced from the host Institute (FBRI of GARES, Jinju-si, Republic of Korea). The germplasm comprised 16 standards, 70 sprays, and 68 pot types, totaling 154 accessions. The list of 154 accessions and their LN regeneration is in supplementary Table S1 (List of chrysanthemum accessions and regeneration of cryopreserved varieties in 2019–2021). For the in vitro introduction of in vivo cuttings, shoot tips were surface sterilized in 75% ethyl alcohol for 30 s and 0.5% sodium hypochlorite (NaOCl) for 15 min with the aid of an oil-type vacuum pump (ULVAC Technologies, Inc., Methuen, MA, USA) and washed with sterilized distilled water 3–4 times, followed by subcultures. In vitro plantlets, in vitro-introduced by the FBRI, and our group were multiplicated via nodal sections through the sequential subcultures by applying a liquid overlay 10–14 days after the inoculation. The subculture medium and conditions for multiplication were similar to the standard subculture described in Section 4.1.

After the multiplication with standard subculture conditions, node-cutting-derived shoot tips were subjected to the droplet-vitrification procedure described previously. Ten to 12 shoot tips/vials by ten cryovials were stored for long-term storage, and an additional two were used for recovery monitoring. The cryopreserved samples were transferred by car to the LN storage facilities at the National Agrobiodiversity Center.

We identified in vitro donor plants of lower LN regeneration varieties, such as var. ‘Purple pearl’ and var. ‘Orange cap’ grew slowly compared to the other accessions. To revitalize the vigorous growth of donor plantlets, we inoculated apical sections with 3–4 nodes instead of single nodal sections. After 10–14 days, liquid MSF was added to the gellan gum-gelled medium, as described in Section 4.1. After 1–2 cycles of apical sections + liquid overlay, LN regeneration of revitalized samples was compared with the first round data (Figure 6).

### 4.4. Recovery Assessment and Statistical Analysis

Survival was evaluated two weeks following cryopreservation by counting the number of shoot tips showing regrowth of greenish tissues. Regeneration was determined after six weeks when the shoots had developed into normal plantlets (≥10 mm) with fully expanded leaves and roots, without either the lag phase or callus formation. Ten to 12 shoot tips were used per experimental condition, and the experiments were replicated no less than twice. Data from all experiments were analyzed by analysis of variance (ANOVA) and Least Significant Difference (LSD) test or Duncan’s multiple range test (DMRT, *p* < 0.05) using SAS (SAS Institute Inc., Cary, NC, USA). Results are presented as percentages with their standard deviations.

## Figures and Tables

**Table 1:** Summary of cryobanking of chrysanthemum collection using the droplet-vitrification protocol in 2019–2021.

Variety Type	No. of Accessions	LN Survival (%)		LN Regeneration (%)	
		Range	Mean	Range	Mean
Standard	16	60.0~100.0	85.9 ± 12.0	50.0~94.4	75.7 ± 12.2
Spray	70	62.5~100.0	83.9 ± 9.3	50.0~100.0	75.0 ± 10.1
Pot	68	61.9~100.0	85.5 ± 9.4	44.4~96.7	74.3 ± 11.6
Total	154		84.9		74.8

The list of 154 accessions and their LN regeneration is available in Supplement (Table S1. List of chrysanthemum accessions and regeneration of cryopreserved varieties in 2019–2021).

**Table 2:** Set of treatments to test the impact of pre-LN, LN, and post-LN procedures on the growth of cryopreserved chrysanthemum shoot tips.

Procedure	Treatment Conditions	Code
Preculture	No-preculture	No-PC
	10% sucrose, 48 h	S-10%
	10% sucrose, 31 h → 17.5% sucrose, 16 h	S-10% → S-17.5%, standard ****
	10% sucrose, 31 h → 25% sucrose, 16 h	S-10% → S-25%
Vitrification solutions	A1-73.7% (PVS2) * ice, 60 min	PVS2 (60 m)
	A3-90% ice, 60 min	A3-90% (60 m)
	A3-80% ice, 60 min	A3-80% (60 m), standard
	A3-80% ice, 40 min	A3-80% (40 m)
	B1-100 (PVS3) 25 °C, 60 min	PVS3 (60 m)
	B5-85% 25 °C, 60 min	B5-85% (60 m)
Osmoprotection, cooling/warming container, regrowth medium	No-osmoprotection, Aluminum foil strips, RM1-RM2-MSF **	No-OP
	C4-35% 30 min, Aluminum foil strips, RM1-RM2-MSF	OP/foil/RM1, standard
	C4-35% 30 min, Cryovial (2 mL), RM1-RM2-SMF	Vial
	C4-35% 30 min, Aluminum foil strips, RM2-RM2-MSF	RM2-RM2-MSF
Regrowth media	NH_4_NO_3_-free + GA1 + BA1 → GA1 + BA1 → MSF ***	−a+h/+h/−h, standard
	NH_4_NO_3_-free + GA1 + BA1 → MSF → MSF	−a+h/−h/−h
	NH_4_NO_3_-free + MSF → GA1 + BA1 → MSF	−a−h/+h/−h
	NH_4_NO_3_-free + MSF → MSF → MSF	−a−h/−h/−h
	NH_4_NO_3_-containing + GA1 + BA1 → GA1 + BA1 → MSF	+a+h/+h/−h
	NH_4_NO_3_-containing + MSF → MSF → MSF	+a-h/−h/−h
	NH_4_NO_3_-free + GA1 + BA1 → GA1 + BA1 → GA1 + BA1	−a+h/+h/+h
	NH_4_NO_3_-containing + GA1 + BA1~ (without change)	+a+h~

* A1-73.7% (PVS2), 30% glycerol + 15% DMSO + 15% EG + 13.7% sucrose, *w/v*); A3-90%, 37.5% glycerol + 15% DMSO + 15% EG + 22.5% sucrose, *w/v*; A3-80%, 33.3% glycerol + 13.3% DMSO + 13.3% EG + 20.1% sucrose, *w/v*; B1-100% (PVS3), 50% glycerol + 50% sucrose, *w/v*; B5-85%, 42.5% glycerol + 42.5% sucrose, *w/v*; C4-35%, 17.5% glycerol + 17.5% sucrose, *w/v*; DMSO, dimethyl sulfoxide; EG, ethylene glycol; The lower concentrated PVSs (A3-80%, B5-85%) were formulated by individual weighing of cryoprotectants, instead of dilution of higher concentrated PVSs (A3-90%, B1-100%). ** RM1 (MS + NH_4_NO_3_-free + GA1+BA1), 5d, dark → RM2 (MS + NH_4_NO_3_-containing + GA1+BA1), 3w2d, 1 L → MSF (MS + growth hormones-free), 2w, 2 L; GA1+BA1, 1 mg L^−1^ gibberellic acid (GA_3_) + 1 mg L^−1^ benzyl adenine (BA). *** MS + NH_4_NO_3_-free/containing + with/without growth hormones, 5d, dark → MS + NH_4_NO_3_-containing + with/without growth hormones, 3w2d, 1L → MSF (MS + with/without growth hormones), 2w, 2 L; 1 L and 2 L, light provided by 1 and 2 fluorescent lamps (40 and 60 µE m^−2^ s^−1^, respectively). **** standard indicates treatments composing the standard procedure where other stages are the same as in the standard protocol.

## Data Availability

Not applicable.

## Supplementary Materials

The following supporting information can be downloaded at: https://www.mdpi.com/article/10.3390/plants12051059/s1, Table S1: List of chrysanthemum accessions and regeneration of cryopreserved varieties in 2019–2021.
