# Supplementary material for: Alternative Plant Vitrification Solution A3-80% and Initial Ammonium-Free Regrowth Medium Enable Cryobanking of Chrysanthemum Germplasm
Source: Plants (Basel). 2023 Feb 27;12(5):1059. doi: 10.3390/plants12051059 (PMC10005143; doi:10.3390/plants12051059)
Supplement: Supplementary file 1 [file plants-2179581-supplementary.pdf › plants-2179581-supplementary.pdf]

Table S1. List of chrysanthemum accessions and regeneration of cryopreserved varieties in 2019-2021.

No.	Year	Type	Accession No.	Variety Name	LN_surv(%)	LN_rege(%)
1	2019	pot	321686	Gaya baby	76.7	80.0
2	2019	pot	321695	Golden pearl	86.7	60.0
3	2019	spray	299603	Green diamond	85.7	66.7
4	2019	spray	321717	Green sense	85.7	71.4
5	2019	spray	321718	Green pasta	77.3	75.0
6	2019	spray	321671	Noble wine	92.0	76.0
7	2019	standard	321691	Danmi	88.2	76.5
8	2019	spray	321636	Dorothy	85.7	85.7
9	2019	spray	321719	Lime farm	85.7	64.3
10	2019	spray	281240	Magics	87.5	87.5
11	2019	spray	321720	Morning angel	77.8	-**
12	2019	standard	310045	Baekgang	60.0	50.0
13	2019	spray	321722	Bravo hope	63.6	63.6
14	2019	spray	321723	Vivace	95.5	77.3
15	2019	standard	321644	Sunmi	88.2	88.2
16	2019	standard	321733	Seolsong	91.7	91.7
17	2019	spray	321724	Sugar cream	81.8	81.8
18	2019	pot	321678	Snow beam	82.8	52.6
19	2019	standard	245232	Shinshinma	78.3	69.6
20	2019	spray	321726	Sunny farm	93.3	80.0
21	2019	spray	281236	Amber	92.6	77.8
22	2019	spray	321679	Elsa	90.0	90.0
23	2019	spray	309230	Yes ruby	88.9	88.9
24	2019	spray	309232	Yes holic	90.0	80.0
25	2019	spray	309233	Yellow cap	-	-
26	2019	spray	321680	Orange cap	62.5	50.0
27	2019	spray	321728	Orange farm	82.6	65.2
28	2019	spray	281237	Joy pink	91.7	91.7
29	2019	pot	321689	Corona yellow	80.0	72.0
30	2019	pot	321674	Corona pink	70.6	55.9
31	2019	spray	321650	Kids orange	88.5	76.9
32	2019	spray	321635	Purple cap	76.5	70.6
33	2019	pot	321693	Purple pearl	66.7	44.4
34	2019	pot	321696	Peach egg	70.8	62.5
35	2019	spray	309265	Field green	64.3	64.3
36	2019	spray	321730	Pink farm	70.0	70.0

37	2019	pot	321676	Harmony red	81.0	72.8	
38	2019	pot	321682	Harmony pink	86.2	79.3	
39	2019	spray	321731	Honey pink	81.0	81.0	
40	2019	spray	321732	White farm	80.0	80.0	81.5
41	2020	pot	281242	Breeze	100.0	96.7	
42	2020	pot	281243	Trumpf yellow	78.3	66.1	
43	2020	pot	321662	Gaya neon	80.0	66.7	
44	2020	pot	321670	Gaya sunshine	68.3	68.3	
45	2020	spray	-*	Golden eye	80.0	73.3	
46	2020	pot	246228	Green valley	83.8	69.4	
47	2020	spray	234217	Green witch	84.2	84.2	
48	2020	spray	-	Daumi	88.9	72.2	
49	2020	spray	234155	Dublin	73.3	60.0	
50	2020	pot	244568	Day dream	91.7	88.7	
51	2020	spray	321664	Dimple	92.9	71.4	
52	2020	spray	-	Lovely song	70.0	60.0	
53	2020	pot	281241	Red wing	75.0	68.3	
54	2020	spray	234159	Lolly farm	85.8	66.7	
55	2020	spray	321669	Magic ballade	81.3	68.8	
56	2020	spray	321721	Moulin rouge	93.3	73.3	
57	2020	standard	245257	Biarritz	84.3	67.1	
58	2020	pot	244573	By minipot	80.1	67.6	
59	2020	spray	234102	Viking	83.3	77.8	
60	2020	spray	-	Bodre	73.3	73.3	
61	2020	spray	-	Bodmi	93.3	86.7	
62	2020	spray	-	Bolkar red	70.0	60.0	
63	2020	spray	321663	Blue hope	100.0	100.0	
64	2020	pot	234123	Visalia salmon	96.4	90.1	
65	2020	spray	321668	Biz	78.9	60.0	
66	2020	spray	-	Saebomi	85.5	75.9	
67	2020	pot	321692	Shine pearl	61.9	58.3	
68	2020	standard	321658	Seolmi	100.0	87.5	
69	2020	spray	234208	Serafina	62.5	58.3	
70	2020	standard	234189	Seinonami	80.0	73.3	
71	2020	standard	246223	Sumi	100.0	94.4	
72	2020	spray	321725	Snow sense	68.3	68.3	
73	2020	standard	246234	Shiosai	92.3	61.5	
74	2020	standard	281234	Eye green	67.7	70.1	
75	2020	spray	321672	Eye ball	84.6	84.6	

76	2020	spray	234156	Yen	84.7	73.9	
77	2020	spray	321727	Angel eye	81.7	70.0	
78	2020	spray	-	Yes nouri	87.5	81.3	
79	2020	pot	321690	Orange egg	80.0	80.0	
80	2020	spray	321657	Orange kids	90.9	63.6	
81	2020	pot	321694	Orange pearl	80.0	80.0	
82	2020	spray	234210	Water fog	83.3	66.7	
83	2020	spray	234129	Euro white	100.0	73.3	
84	2020	pot	244567	Jagalro	86.4	85.7	
85	2020	spray	321649	Joy cream	88.9	94.4	
86	2020	pot	244561	Chesapeake	100.0	93.3	
87	2020	pot	321647	Cristal red	85.7	78.6	
88	2020	spray	234182	Two tone	100.0	93.8	
89	2020	pot	321675	Point egg	65.0	60.0	
90	2020	spray	234131	Progi	87.3	75.4	
91	2020	spray	-	Prima pink	68.3	67.5	
92	2020	spray	321665	Fiore	81.7	74.2	
93	2020	pot	244575	Poico Fun	93.3	80.0	
94	2020	pot	244566	Pixie Time Pink	80.9	66.6	
95	2020	spray	234158	Feeling green	90.0	85.0	
96	2020	pot	321685	Pink egg	75.0	80.0	
97	2020	spray	-	Pink pride	66.7	58.3	
98	2020	spray	234207	Pinky	93.3	83.3	
99	2020	pot	321684	Harmony purple	86.7	-	
100	2020	spray	244560	Hirario	97.2	87.3	83.7
101	2021	pot	281244	Trumpf violet	100.0	69.2	
102	2021	pot	321613	Gaya gold	93.8	76.8	
103	2021	pot	246226	Gaya glory	89.5	76.3	
104	2021	pot	321660	Gaya nano	73.3	60.0	
105	2021	pot	321640	Gaya dream	87.8	75.6	
106	2021	pot	234224	Gaya red	70.0	-	
107	2021	pot	324642	Gaya luna	92.2	78.0	
108	2021	pot	281246	Gaya ruby	100.0	91.7	
109	2021	pot	321653	Gaya rouge	92.9	69.0	
110	2021	pot	321641	Gaya sunset	80.1	74.2	
111	2021	pot	321661	Gaya sense	93.3	93.3	
112	2021	pot	246219	Gaya sun	86.7	66.7	
113	2021	pot	246225	Gaya art	91.2	78.5	
114	2021	pot	234228	Gaya orange	88.7	70.3	

115	2021	pot	321687	Gaya purple	89.1	76.0
116	2021	pot	234230	Gaya white	82.5	66.3
117	2021	pot	281248	Gaya hit	86.4	73.5
118	2021	pot	321655	Golden egg	100.0	100.0
119	2021	pot	246222	Golden tree	88.9	-
120	2021	spray	321666	Green flora	83.3	66.7
121	2021	pot	244571	Dark free poly	85.0	65.0
122	2021	spray	245255	Delhi prime	86.7	66.7
123	2021	pot	281249	Lemon eyes	83.1	76.8
124	2021	spray	244553	Rocky	87.8	70.0
125	2021	spray	244548	Rerence	87.5	81.3
126	2021	pot	244564	Liz way	77.7	70.3
127	2021	pot	244562	Little rock	84.1	74.9
128	2021	pot	244563	Madras	80.0	70.0
129	2021	spray	321656	Magic star	90.0	80.0
130	2021	pot	244572	Veria dark	95.0	90.0
131	2021	spray	234132	Vesuvio white	95.8	83.3
132	2021	spray	245256	Biarritz pink	85.7	76.2
133	2021	standard	234191	Seino makoto	83.8	72.5
134	2021	standard	234190	Seino kyoku	93.3	80.0
135	2021	spray	321677	Sun green	86.7	73.3
136	2021	pot	244576	Amarillo	100.0	95.0
137	2021	standard	327372	Yonglong	76.7	65.8
138	2021	spray	327369	Yes mimi	93.8	87.5
139	2021	standard	281239	Euro yellow	89.6	82.1
140	2021	standard	246235	Yucca	100.0	80.0
141	2021	spray	246230	Jembla	83.3	75.0
142	2021	pot	234229	G&B	91.7	66.7
143	2021	pot	327168	Corona heart	85.0	60.0
144	2021	pot	321645	Cristal ruby	100.0	80.0
145	2021	pot	321646	Cristal bronze	85.0	75.0
146	2021	pot	246221	Purple tree		-
147	2021	pot	-	Purple pink	86.7	80.0
148	2021	pot	321654	Pearl egg	86.7	73.3
149	2021	pot	244574	Pico amor	90.0	70.0
150	2021	pot	234220	Pink eyes	90.0	76.7
151	2021	pot	321681	Harmony yellow	92.0	79.5
152	2021	pot	321688	Harmony white	100.0	70.0
153	2021	pot	246220	Happy eye	93.3	93.3

154	2021	pot	234226	White angel	90.0	-	88.8
			Mean		**84.9**	**74.8**	

* registeration number is not available yet.

** survival and regeneration data were not recorded in the excel form, possible due to the contamination and miss

Variety name was translated from Korean to English.

In 2021, six more accessions of sweet potato germplasm was cryopreserved, but not included in this table.

72.9

74.9

76.0

sing.
